# Towards an actionable One Health approach

**DOI:** 10.1186/s40249-024-01198-0

**Published:** 2024-04-12

**Authors:** Xiao-Xi Zhang, Zohar Lederman, Le-Fei Han, Janna M. Schurer, Li-Hua Xiao, Zhi-Bing Zhang, Qiu-Lan Chen, Dirk Pfeiffer, Michael P. Ward, Banchob Sripa, Sarah Gabriël, Kuldeep Dhama, Krishna Prasad Acharya, Lucy J. Robertson, Sharon L. Deem, Cécile Aenishaenslin, Filipe Dantas-Torres, Domenico Otranto, Delia Grace, Yang Wang, Peng Li, Chao Fu, Patrícia Poeta, Md. Tanvir Rahman, Kokouvi Kassegne, Yong-Zhang Zhu, Kun Yin, Jiming Liu, Zhao-Jun Wang, Xiao-Kui Guo, Wen-Feng Gong, Bernhard Schwartländer, Ming-Hui Ren, Xiao-Nong Zhou

**Affiliations:** 1 School of Global Health, Chinese Center for Tropical Diseases Research, Shanghai Jiao Tong University School of Medicine, Shanghai, People’s Republic of China; 2 Institute of One Health, Shanghai Jiao Tong University, Shanghai, People’s Republic of China; 3 Medical Ethics and Humanities Unit, Hong Kong University, Hong Kong, People’s Republic of China; 4 Center for One Health, University of Global Health Equity, Butaro, Rwanda; 5 College of Veterinary Medicine, South China Agricultural University, Guangzhou, People’s Republic of China; 6 School of Ecology and Environment, Hainan University, Haikou, Hainan People’s Republic of China; 7 Branch of animal and vector-borne diseases, Division of Infectious Disease Control, Chinese Center for Disease Control and Prevention, Beijing, People’s Republic of China; 8 Centre for Applied One Health Research and Policy Advice, Department of Infectious Diseases and Public Health, Jockey Club College of Veterinary Medicine and Life Sciences, City University of Hong Kong, Hong Kong SAR, People’s Republic of China; 9 Department of Pathobiology and Population Sciences, Royal Veterinary College, London, United Kingdom; 10 Sydney School of Veterinary Science, The University of Sydney, Camden, Australia; 11 Tropical Disease Research Center, Department of Tropical Medicine, Faculty of Medicine, Khon Kaen University, Khon Kaen, Thailand; 12 Laboratory of foodborne parasitic zoonoses, Department of translational physiology, infectiology and public health, Chair Faculty Committee on Internationalisation, Faculty of Veterinary Medicine, Ghent University, Merelbeke, Belgium; 13 Division of Pathology, ICAR-Indian Veterinary Research Institute (IVRI), Bareilly, Uttar Pradesh India; 14 Department of Livestock Services, Animal Quarantine Office-Kathmandu, Budhanilkantha, Kathmandu, Nepal; 15 Parasitology, Department of Paraclinical Sciences, Faculty of Veterinary Medicine, Norwegian University of Life Sciences, Ås, Norway; 16 One Government Drive, Saint Louis Zoo Institute for Conservation Medicine, St. Louis, USA; 17 Groupe de Recherche en Épidémiologie des Zoonoses et Santé Publique (GREZOSP), Faculté de médecine vétérinaire, Université de Montréal, Saint-Hyacinthe, Québec Canada; 18 Centre de recherche en santé publique de l, Université de Montréal et du CIUSSS du Centre-Sud-de-l’Île-de-Montréal, Montréal, Québec Canada; 19 Department of Immunology, Aggeu Magalhães Institute, Oswaldo Cruz Foundation (Fiocruz), Recife, Brazil; 20 Department of Veterinary Medicine, University of Bari, Valenzano, Italy; 21 Department of Pathobiology, Faculty of Veterinary Science, Bu-Ali Sina University, Hamedan, Iran; 22 Natural Resources Institute, University of Greenwich, Chatham Maritime, UK; 23 International Livestock Research Institute, Nairobi, Kenya; 24 Director of Key Laboratory of Animal Antimicrobial Resistance Surveillance, Ministry of Agriculture and Rural Affairs, Beijing, People’s Republic of China; 25 Institute of Animal Science, Chinese Academy of Agricultural Sciences, Beijing, People’s Republic of China; 26 Key Laboratory of Ecosystem Network Observation and Modeling, Institute of Geographic Sciences and Natural Resources Research, Chinese Academy of Sciences, Beijing, People’s Republic of China; 27 United Nations Environment Programme-International Ecosystem Management Partnership (UNEP-IEMP), Beijing, People’s Republic of China; 28 Microbiology and Antibiotic Resistance Team, Department of Veterinary Sciences, University of Trás-os-Montes and Alto Douro, Vila Real, Portugal; 29 Associate Laboratory for Green Chemistry, Chemistry Department, University Nova of Lisbon, Lis-bon, Portugal; 30 Department of Microbiology and Hygiene, Bangladesh Agricultural University, Mymensingh, Bangladesh; 31 Faculty of Science, Hong Kong Baptist University, Hong Kong SAR, People’s Republic of China; 32 The Bill &, Melinda Gates Foundation, Seattle, WA USA; 33 German Ministry of Foreign Afairs (Former Assistant Director General and Chef de Cab‑inet of Dr Tedros at the World Health Organization), Berlin, Germany; 34 School of Public Health, Peking University, Beijing, People’s Republic of China; 35 National Institute of Parasitic Diseases at Chinese Center for Disease Control and Prevention (Chinese Center for Tropical Diseases Research), NHC Key Laboratory of Parasite and Vector Biology, WHO Collaborating Centre for Tropical Diseases, Shanghai, People’s Republic of China

**Keywords:** One Health, Global Health, One Health Action Commission, Research agenda

## Abstract

**Background:**

Despite the increasing focus on strengthening One Health capacity building on global level, challenges remain in devising and implementing real-world interventions particularly in the Asia-Pacific region. Recognizing these gaps, the One Health Action Commission (OHAC) was established as an academic community for One Health action with an emphasis on research agenda setting to identify actions for highest impact.

**Main text:**

This viewpoint describes the agenda of, and motivation for, the recently formed OHAC. Recognizing the urgent need for evidence to support the formulation of necessary action plans, OHAC advocates the adoption of both bottom-up and top-down approaches to identify the current gaps in combating zoonoses, antimicrobial resistance, addressing food safety, and to enhance capacity building for context-sensitive One Health implementation.

**Conclusions:**

By promoting broader engagement and connection of multidisciplinary stakeholders, OHAC envisions a collaborative global platform for the generation of innovative One Health knowledge, distilled practical experience and actionable policy advice, guided by strong ethical principles of One Health.

**Graphical Abstract:**

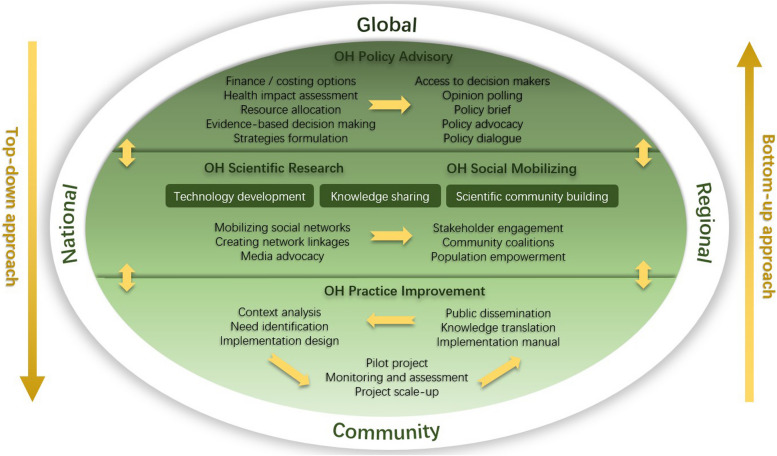

## Background

Global ecosystems are under serious threat by a changing climate, as demonstrated by increasing episodes or severity of extreme events, such as floods, droughts, glacial ablation, rising sea levels and prolonged sand storms in recent years. Rapid urbanization, agricultural intensification and other anthropogenic actions have fundamentally altered the natural habitats of wildlife, as well as affected humans interact with natural ecosystems. The COVID-19 pandemic has clearly demonstrated the need for intersectoral action to address emerging health threats at the nexus of human, animal and the environment [1, 2].

The establishment of the Quadripartite in 2022 is a milestone that demonstrates political commitments to the promotion of One Health (OH) with a truly holistic perspective on global ecosystems. Perhaps for the first time in history, the One Health High Level Expert Panel (a scientific advisory body to the Quadripartite), working under the auspices of several major international health organizations, has made an explicit commitment and called for a “socioecological equilibrium that seeks a harmonious balance between human-animal-environment interaction and acknowledging the importance of biodiversity, access to sufficient natural space and resources, and the intrinsic value of all living things within the ecosystem” [3].

However, if such statements are not embedded in a rigorous ethical framework or do not result in actual implementation of interdisciplinary scientific research that will lead to policy-making based on OH thinking, they will remain empty and likely undermine the influence and credibility of the organisations that issues them. Beyond ethics, challenges remain in devising and implementing real-world interventions aligned with the targets and goals set by the Quadripartite [4]. National and international coordinating mechanisms are still unable to effectively enact intersectoral collaboration, and professional and sectoral silo mentality is still the rule rather than the exception. Recognizing these gaps, the One Health Action Commission (OHAC) was established.

### The one health action commission

The OHAC is comprised of experts from various disciplines around the world. Fifty experts were initially identified through an established database of OH expertise [5], according to their academic records and impacts, with a geographical focus on the Asia-Pacific region. They were invited to participate in the first OHAC workshop, organized in Shanghai on May 8–10, 2023, with support from the Bill & Melinda Gates Foundation and hosted by Shanghai Jiao Tong University One Health Institute. A scientific steering committee was elected during the workshop and an initial research agenda was identified.

**Fig. 1:**
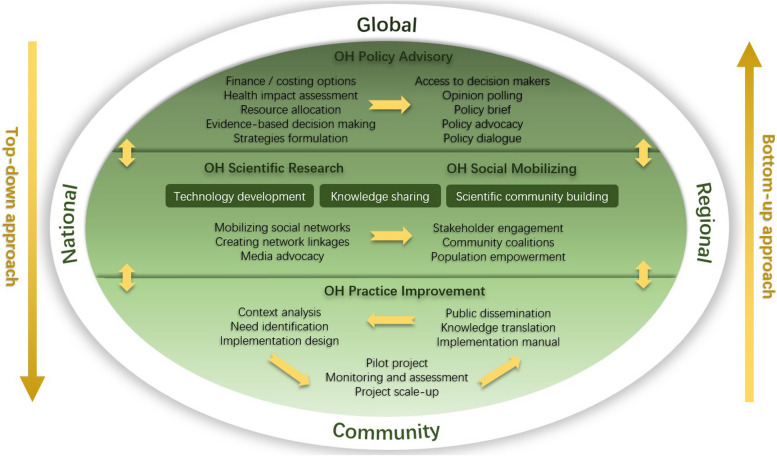
The application of bottom-up and top-down approaches for One Health promotion advocated by One Health Action Commission. *OH* One Health

The areas that distinguish OHAC from other OH groups are threefold. First, OHAC will target its efforts on OH action at the national level with a focus on the Asia-Pacific region. This is a reflection of current risks for pandemic threats present in the Asia-Pacific region. Second, members of OHAC recognize the importance of health policies that are embedded within internationally agreed ethical frameworks. If OH approaches are indeed distinct from more traditional public health approaches, it is through their re-orientation towards the interests of the whole human-animal-environment complex system. Such interests, in turn, yield duties on the part of humans as the main moral agents. Third, the OHAC acknowledges the importance of research and action that respond to acute and long-term needs, highlighting the importance of both top-down and bottom-up approaches in OH research, particularly on social science and economic considerations (Fig. 1).

In 2022, the Quadripartite developed the One Health Joint Plan of Action (2022–2026) (OHJPA) and an implementation guide is also under development. According to the action tracks for OH promotion proposed in the OHJPA, OHAC has set the following research priorities, in order to focus the planning activities and resources, which also align with its scientific and practical expertise and focus on the Asia-Pacific region.

### Zoonoses control strategies at the interface of human-animal-environment

Four priorities identified by OHAC to improve the zoonoses control strategies. Firstly, it is essential to put more emphasis on the monitoring and prevention strategies of wildlife diseases, since a vast number of wildlife species which in turn harbor a huge number of pathogen species is making more difficulties to identify, monitor, forecast the risk of wildlife diseases. Secondly, it is essential to investigate more on complexity of zoonotic spillover mechanisms, due to drive forces of zoonotic spillover are unclear up to now, and integrating an ecological, biomedical and social science lens into research could help elucidate the complex interplay of factors that drive disease dynamics in different eco-social contexts. Thirdly, risks to humans are of course the main concern of most relevant stakeholders, but a robust OH approach should also consider the wellbeing of animals and the sustainability of the natural environment as of fundamental importance, both in themselves and due to their intrinsic association with human wellbeing and sustainability of our ecosystems. Fourthly, more international initiatives are encouraged to fill in the gaps in studies, monitoring and prevention of diseases between in humans, domestic and wild animals in certain ecosystems, following the steps of a Wildlife Disease Research Network (WDRN), co-sponsored by International Zoological Society worked together with the International Union of Biological Sciences, which have launched recently.

### Risk mapping of antimicrobial resistance hotspots

The application of a OH approach in both understanding and designing interventions and policies to combat antimicrobial resistance is necessary with following four priorities identified by OHAC. Firstly, it is essential to perform the risk mapping to identify hot spots of antimicrobial resistance (AMR) that can guide workable interventions by identifying the areas or populations that are most affected by – or that contribute most to – the spread of resistant microorganisms [6]. For example, urban informal settlements in low- and middle-income countries (LMICs) might become hot spots of AMR due to the high density of humans and animals, the unregulated use of antibiotics in both human and animal treatments, and the lack of adequate water and sanitation infrastructure [7]. Secondly, the interests of farmers and their economic wellbeing should also be considered to associate the risk changes of AMR, particularly in LMICs. Thirdly, geospatial analyses and data visualization techniques can be employed in the risk mapping to identify potential hot spots of AMR, such that variations in antimicrobial susceptibility rates can be investigated at the local/ neighborhood level. Fourthly, proper dissemination of risk mapping is encouraged to promote the antimicrobial stewardship and judicious by balancing farmers’ interests with the risks of AMR, e.g., by better community-based education campaigns and improvement of sanitation infrastructure.

### Food security and food safety hazards associated with animal-derived food

Environmental, animal, and human interests will have to be appropriately balanced to allow human and animal communities to flourish within a sustainable global ecosystem, with following priority actions identified by OHAC. Firstly, special measures should be directed towards transmission pathways for both diseases and AMR, including understanding more how these pathways can be managed and implementing in both land-animal production and aquaculture, along with the national food security program in intensifying their animal or animal-based food production in different countries. Secondly, capacity-building can focus on enhancing the skills and knowledge to identify and control the factors that contribute to food safety hazards, with a particular emphasis on small-scale farmers. Thirdly, all capacities to identify and tackle the risks in food security and food safety in food production chain are necessary to be improved.

### Innovations in surveillance for effective control of pandemics/ epidemics/ endemics

The establishment and maintenance of OH databases, in which data are collected from human, animals and the environment, are facing challenges significantly in the surveillance of diseases control [8]. It is difficult to monitor the process and quality of surveillance if the OH database is not well collation with the joint databases from different sectors, whereby action in human populations can be based on a threshold set in animal or environment samples. Examples include COVID surveillance in wastewater that enabled attention to specific communities where peaks were detected, and prevalence of *Taenia solium* in pigs as the marker for initiating mass drug administration in humans.

The remarkable breakthroughs made in the application of affordable, accurate, portable point-of-care diagnostic platforms during COVID-19 have brought about obvious possibilities for a new model of operation for surveillance. With the advances of point-of-care diagnostic technologies, surveillance points are being deployed to the front line of application, such as villages, fields, farms, and instantly transmitted using data technologies which then inform rapid decision in practice. Meanwhile, the adoption of artificial intelligence (AI) should be further explored for spatio-temporal pandemic risk assessment, which will be beneficial to optimizing resource allocation in handling the public health emergences to reduces the economic burden of epidemics.

### Prototype/ best practice research to distill and disseminate experiences in national and regional context

While OH practice is not new and there are many examples, we are in a stage of moving OH theory into practice at many levels, from the most local to the highest intersectoral policy development, and demonstrating and highlighting good practice at a range of levels is incredibly important. As we come to understand the successes and failures of the global response to COVID-19, the needs for formulating context-sensitive local strategies and distilling best practices have never become more critical. Prototype/ best practice research is needed to support and translate the OH concept and high-level activities into practical guidance that can be adapted to national level contexts, with supported by economic evaluation approaches.

For instance, the experiences and lessons learnt from China’s national schistosomiasis control and elimination programs are an example of an OH prototype. In Asia and the Pacific region, there are a wealth of examples that can potentially be developed into OH best practices. So far, OH approaches have been applied in the management of avian influenza H7N9 in China [9], Nipah virus infections in Bangladesh [10], and human liver fluke in Thailand [11], etc. Additionally, regional platforms, such as Bangladesh Country Coordination Mechanism (BCCM) established in 2002, and the Southeast Asia One Health University Network (SEAOHUN) established in 2011, have substantially contributed to building a competent OH workforce in Southeast Asia.

### Laying the foundations with bottom-up and top-down approaches

Adopting a OH approach is not meant to create collaboration for the sake of collaboration; the aim is to solve problems and create efficiencies. Potential trade-offs, misalignments, and conflicts of interest should be recognized and understood as early as possible to allow for identification of possible solutions. OHAC is committed to the application of both bottom-up and top-down approaches (Fig. 1):

(i) A bottom-up approach that aims to understand the needs of OH action in specific localities, and to identify action points for OH implementation at the community level. OH is a highly context-sensitive approach as it is closely related to local natural and social conditions. OH work thus necessitates in-depth assessment and understanding of interests before OH activities are started or even planned. Meanwhile, when applying OH strategies to real-world scenarios, special attention must be given to how scientific knowledge can be translated into information that can be understood, accepted, and finally, owned by local residents. Greater participation of local, regional and national stakeholders from the very start of the process is essential to obtain community and political engagement for OH policies [12].
(ii) A top-down approach to coordinate the use of resources to translate global, regional and national goals into local actions. Integration of OH approaches into higher level decision-making processes will require a stronger evidence-base in support of the economic impact of OH policies such as the economics for biodiversity by the Dasgupta review [13]. Innovative mechanisms and actions are urgently required to connect global agenda with the local situation. Meanwhile, there is also a need to develop and adapt frameworks and tools to evaluate the implementation of OH actions at various levels. Large-scale data-based evaluation tools, such as the global One Health index [14, 15], are necessary to identify global patterns in general community needs.

## Conclusions

The launch of the Quadripartite OHJPA and the preparation of its implementation guide, indicate the growing efforts at the global level towards translating OH policies into real-world changes. With the vision of building an international community committed to the OH systems perspective, OHAC aims to actively respond to global needs to promote health sustainability through a OH approach, thereby also preventing future pandemics, combatting AMR, supporting the development of safe and sustainable food systems and more. The time to act is now.

## Data Availability

The datasets used and/or analysed during the current study are available from the corresponding author upon reasonable request.

## Abbreviations

AI: Artificial intelligence
AMR: Antimicrobialresistance
BCCM: Bangladesh Country Coordination Mechanism
LMICs: Low- and middle-income countries
NiV: Nipah virus
OH: One Health
OHAC: One Health Action Commission
OHJPA: One Health Joint Plan of Action
SEAOHUN: Southeast Asia One Health University Network
WDRN: Wildlife Disease Research Network

## References

[1] Ward MP (2020). SARS-CoV-2, where to now?. Transbound Emerg Dis.

[2] Zhang X-X, Jin Y-Z, Lu Y-H, Huang L-L, Wu C-X, Lv S (2023). Infectious disease control: from health security strengthening to health systems improvement at global level. Glob Health Res Policy.

[3] World Health Organization. One Health High-Level Expert Panel (OHHLEP): World Health Organization; 2023. Available from: https://www.who.int/groups/one-health-high-level-expert-panel/meetings-and-working-groups.

[4] The Food and Agriculture Organization of the United Nations, the United Nations Environment Programme, the World Health Organization, and the World Organization for Animal Health. One health joint plan of action (2022‒2026): working together for the health of humans, animals, plants and the environment. Geneva: World Health Organization; 2022.

[5] Qiang N, Gu SY, Wang XY, Zhang XX, Xia S, Zheng JX (2022). A One Health information database based on standard bibliometric analysis. Sci One Health.

[6] Zhang XX, Li XC, Zhang QY, Liu JS, Han LF, Lederman Z (2023). Tackling global health security by building an academic community for One Health action. Infect Dis Poverty.

[7] Nadimpalli ML, Marks SJ, Montealegre MC, Gilman RH, Pajuelo MJ, Saito M (2020). Urban informal settlements as hotspots of antimicrobial resistance and the need to curb environmental transmission. Nat Microbiol.

[8] Liu JS, Li XC, Zhang QY, Han LF, Xia S, Kassegne K, et al. China's application of the One Health approach in addressing public health threats at the human-animal-environment interface: advances and challenges. One Health. 2023;17:100607. 10.1016/j.onehlt.2023.100607 PMC10425407 37588422

[9] Wu J, Ke C, Lau EHY, Song Y, Cheng KL, Zou L (2019). Influenza H5/H7 virus vaccination in poultry and reduction of zoonotic infections, Guangdong Province, China, 2017–18. Emerg Infect Dis.

[10] Ashraf L (2020). Interventions for prevention of Nipah virus transmission and infection in Bangladesh. Arch Commun Med Publ Hlth.

[11] Kompor P, Karn RM, Norkaew J, Kujapun J, Photipim M, Ponphimai S (2016). Population-based intervention for liver fluke prevention and control in Meuang Yang district, Nakhon Ratchasima Province Thailand. Asian Pac J Cancer Prev.

[12] Agrawal R, Murmu J, Pattnaik S, Kanungo S, Pati S (2023). One Health: navigating plague in Madagascar amidst COVID-19. Infect Dis Poverty.

[13] Dasgupta P (2021). The economics of biodiversity: the Dasgupta review.

[14] Zhang XX, Liu JS, Han LF, Xia S, Li SZ, Li OY (2022). Towards a global One Health index: a potential assessment tool for One Health performance. Infect Dis Poverty.

[15] Zhang XX, Liu JS, Han LF, Simm G, Guo XK, Zhou XN (2022). One Health: new evaluation framework launched. Nature.

